# Microbiological Profile and Antimicrobial Resistance in Intensive Care Unit Patients With Sepsis and Septic Shock: A Prospective Single-Center Study From a Tertiary Care Intensive Care Unit

**DOI:** 10.7759/cureus.107160

**Published:** 2026-04-16

**Authors:** Kaoutar Zirhirhi, Othmane Tahri Joutey, Sara Lamghari, Sara Chabbar, Anass Mounir, Bouhouri Aziz

**Affiliations:** 1 Anesthesiology and Reanimation, Centre Hospitalier Universitaire Ibn Rochd, Casablanca, MAR; 2 Anesthesiology and Critical Care, Ibn Rochd University Hospital, Casablanca, MAR; 3 Intensive Care Unit, University of Hassan II Casablanca, Casablanca, MAR

**Keywords:** antimicrobial resistance, icu microbiology, infection site, mortality, multidrug-resistant organisms, sepsis, septic shock

## Abstract

Background

Sepsis and septic shock remain the major causes of mortality in intensive care units (ICUs), increasingly complicated by antimicrobial resistance. However, microbiological profiles and resistance patterns vary widely across regions, and data from North African ICUs remain limited. This study aimed to describe the distribution of pathogens according to infection site, to analyze antimicrobial susceptibility patterns, and to assess the burden of multidrug-resistant organisms among ICU patients with sepsis and septic shock.

Methods

We conducted a prospective observational study in a mixed medical-surgical ICU at Ibn Rochd University Hospital, Casablanca, Morocco, over a 30-month period (January 2023-June 2025). Adult patients with sepsis or septic shock, defined according to Sepsis-3 criteria, were included. Microbiological analysis was restricted to patients with at least one clinically significant isolate, and only the first isolate per infectious episode was considered. Data included infection sites, pathogen distribution, antimicrobial resistance profiles, and ICU mortality.

Results

Eighty patients were included, yielding 192 microbiological samples, of which 126 (65.6%) were positive, corresponding to 142 clinically significant isolates. Polymicrobial infections were identified in 22/80 patients (27.5%). Gram-negative bacteria predominated overall, with *Acinetobacter *spp. (28/142, 19.7%), *Escherichia coli* (26/142, 18.3%), and *Pseudomonas aeruginosa* (20/142, 14.1%) as the most frequently identified pathogens. Multidrug resistance (MDR) was observed in 58/142 isolates (40.8%) and in 36/80 patients (45%). The highest MDR rates were found in *Acinetobacter *spp. (22/28, 78.6%), which was also the leading pathogen in bloodstream (18/52, 34.6%) and respiratory (12/42, 28.6%) infections. *Acinetobacter *spp. exhibited high resistance rates to ciprofloxacin (20/26, 76.9%), gentamicin (20/23, 87.0%), and imipenem (21/27, 77.8%), while colistin resistance remained low (3/28, 10.7%). The overall ICU mortality rate was 36/80 (45%). MDR infections were significantly associated with higher ICU mortality (24/36 (67%) vs. 12/44 (27%), p < 0.001).

Conclusion

In this North African ICU, sepsis was characterized by a high burden of multidrug-resistant Gram-negative bacteria, with a prominent role of *Acinetobacter* spp. across major infection sites. MDR infections were associated with higher ICU mortality, highlighting the potential clinical impact of antimicrobial resistance in critically ill patients. These findings suggest that empirical antibiotic strategies should be adapted to local ICU ecology, with consideration of multidrug-resistant Gram-negative bacteria in high-risk patients and a site-specific approach.

## Introduction

Sepsis and septic shock remain the major causes of morbidity and mortality in intensive care units (ICUs) worldwide [1]. Their management is increasingly challenging due to the rising prevalence of antimicrobial resistance, which directly affects the adequacy of empirical antibiotic therapy and clinical outcomes [2].

However, the microbiological epidemiology of sepsis is highly variable across regions, healthcare systems, and individual ICUs [3]. Differences in antimicrobial use, infection control practices, and patient populations lead to distinct local ecological patterns. As a result, empirical antibiotic strategies based on international data may be inappropriate in specific settings [4].

This issue is particularly relevant in low- and middle-income countries, where ICU-specific microbiological data remain limited [1,3]. In North African settings, local data on pathogen distribution and antimicrobial resistance remain limited, particularly regarding ICU-specific antibiograms stratified by infection site, prospective post-2020 data, and studies linking microbial ecology to clinical outcomes in critically ill patients. This lack of context-specific evidence may contribute to inappropriate empirical therapy and delays in adequate treatment.

Furthermore, microbiological patterns may vary not only between regions but also within the same ICU, particularly according to the site of infection [5]. Despite this, most studies report overall pathogen distribution without adequately addressing site-specific variations, which are critical for guiding empirical antibiotic choices.

From a clinical perspective, this limitation has important implications. Empirical antibiotic therapy that does not account for both local microbial ecology and infection site is likely to be inadequate, particularly in settings with a high prevalence of multidrug-resistant organisms [2,4]. Such inadequacy may delay effective treatment, increase morbidity and mortality, and contribute to the further selection of resistant pathogens.

Therefore, precise characterization of ICU-specific and site-specific microbiological patterns is essential to guide early, appropriate, and targeted empirical antibiotic therapy.

In this context, the present study aimed to characterize pathogen distribution according to infection site in a tertiary care ICU in Morocco. Secondary objectives included the analysis of antimicrobial susceptibility patterns of major bacterial species and the assessment of the burden of multidrug-resistant organisms.

## Materials and methods

Study design and setting

This was a prospective, single-center observational study conducted in a mixed medical-surgical ICU at Ibn Rochd University Hospital, a tertiary care center in Casablanca, Morocco, over a 30-month period (January 2023-June 2025). The study period was defined a priori to allow sufficient enrollment of patients with microbiologically documented sepsis in a single-center ICU with limited capacity, while capturing potential seasonal and temporal variations in pathogen distribution and antimicrobial resistance patterns. The end date corresponded to the completion of the predefined prospective data collection phase.

Study population

All consecutive adult patients (≥18 years) admitted to the ICU with a diagnosis of sepsis or septic shock during the study period were screened for inclusion in the clinical analysis, including both patients presenting with sepsis at ICU admission and those who developed sepsis during their ICU stay.

Microbiological analyses were restricted to a predefined subgroup of patients with at least one clinically significant isolate. A clinically significant isolate was defined as a pathogen recovered from a clinical specimen in the context of compatible clinical signs of infection, after exclusion of contamination and colonization based on predefined clinical, radiological, and microbiological criteria (see “Infection definitions”).

Patients without microbiological documentation or with incomplete microbiological data were excluded from the microbiological analysis. This may introduce a selection bias toward more severe or microbiologically documented infections and may limit the generalizability of the findings.

Sepsis and septic shock were defined according to the Sepsis-3 criteria [6]. Sepsis was defined as a suspected or documented infection associated with acute organ dysfunction, and septic shock as a subset of sepsis requiring vasopressor support despite adequate fluid resuscitation.

Data collection

Data were collected prospectively using a standardized case report form. Baseline characteristics included demographic data (age, sex), underlying comorbidities (hypertension, diabetes mellitus, chronic kidney disease, and malignancy), type of ICU admission (medical or surgical), origin of infection (community-acquired, hospital-acquired, or ICU-acquired), and severity at ICU admission assessed using the SOFA score and Sepsis-3 criteria. Healthcare-related exposure variables included the use and duration of invasive devices (mechanical ventilation and central venous catheterization), prior antibiotic exposure within 90 days before ICU admission, and prior hospitalization within the preceding three months. Infection-related variables included the timing of sepsis onset and the need for organ support therapies (vasopressors, mechanical ventilation, and renal replacement therapy), as well as ICU length of stay prior to infection onset. Microbiological data included site of infection, number and type of samples, pathogen identification, polymicrobial infections, and antimicrobial resistance profiles. Clinical outcomes included ICU length of stay and ICU mortality.

Microbiological sampling and analysis 

Microbiological samples were obtained at the time of clinical suspicion of infection and, whenever possible, prior to the initiation of antibiotic therapy. Samples included blood cultures (at least two sets per patient), respiratory samples (endotracheal aspirates or protected distal bronchial samples), peritoneal fluid cultures, urine cultures, and catheter-related samples, based on clinical judgment. Bacterial identification was performed using matrix-assisted laser desorption/ionization time-of-flight mass spectrometry (MALDI-TOF). Identification and reporting were carried out at the genus or species level whenever possible, rather than grouping isolates at higher taxonomic levels (e.g., Enterobacterales), in order to ensure precise microbiological characterization. Antimicrobial susceptibility testing was performed using an automated system (VITEK 2, bioMérieux) and interpreted according to the European Committee on Antimicrobial Susceptibility Testing (EUCAST) guidelines (version 2023, breakpoint tables v13.0). For the purpose of resistance analysis, isolates with intermediate susceptibility were categorized as resistant.

Infection definitions

Bloodstream infection was defined as the isolation of a clinically significant microorganism from blood cultures in a patient with compatible clinical signs of infection. Recognized pathogens isolated from at least one blood culture were considered significant when not attributable to another infection site. For common skin commensals, particularly coagulase-negative staphylococci, at least two positive blood cultures drawn on separate occasions, together with compatible clinical findings, were required to distinguish true bloodstream infection from contamination. When a bloodstream isolate could be attributed to another documented infection site, it was classified as secondary; otherwise, it was considered a primary bloodstream infection.

Respiratory infection was diagnosed on the basis of compatible clinical findings, radiological evidence of pneumonia, and supportive microbiological results. Respiratory isolates were considered clinically significant only when interpreted in conjunction with these criteria. Quantitative thresholds were considered when available, including ≥10⁶ CFU/mL for endotracheal aspirates and ≥10³ CFU/mL for protected distal bronchial samples, in order to reduce misclassification between colonization and true infection.

Urinary tract infection was defined as the isolation of a uropathogen at a count of ≥10⁵ CFU/mL from a midstream urine or catheter specimen, in a patient with compatible clinical signs of infection, in the absence of another identified source.

Catheter-related bloodstream infection was diagnosed when the same microorganism was recovered from at least one peripheral blood culture and the catheter tip culture in a patient with compatible clinical signs of infection. When available, paired catheter-drawn and peripheral blood cultures with a differential time to positivity of at least two hours were also considered supportive of the diagnosis.

Potential contaminants were excluded based on combined clinical and microbiological assessment. The distinction between contamination, colonization, and true infection was established using predefined clinical, radiological, and microbiological criteria, and validated through joint assessment by the treating intensivist and the microbiologist.

Regarding the origin of infection, community-acquired infections were defined as those present at or within 48 hours of hospital admission. Hospital-acquired infections were defined as those occurring more than 48 hours after hospital admission, and ICU-acquired infections as those developing more than 48 hours after ICU admission. The latter two categories were grouped for analysis due to the limited sample size, as the primary distinction of interest was between community-acquired and healthcare-associated infections.

Infection classification and analysis

Infectious episodes were defined at the patient level, and only the first isolate per patient per infectious episode was considered to avoid duplication bias.

Polymicrobial infections were defined as the isolation of more than one clinically significant pathogen from the same infectious episode, regardless of whether the isolates were recovered from the same or different sample types. Contaminants and colonizers were excluded prior to this classification based on the predefined criteria detailed in the “Infection definitions” subsection.

Multidrug-resistant organisms were defined as acquired non-susceptibility to at least one agent in three or more antimicrobial categories, according to the international expert proposal by Magiorakos et al. [7].

Analyses were performed at both the patient level and the isolate level. Patient-based analyses were used for clinical characteristics, infection site classification, group comparisons (survivors vs. non-survivors; multidrug resistance (MDR) vs. non-MDR), and outcome assessment, whereas isolate-based analyses were used for pathogen distribution and antimicrobial resistance profiling.

Statistical analysis

Quantitative variables were expressed as mean ± standard deviation or median (interquartile range), depending on data distribution assessed using the Shapiro-Wilk test. Age and SOFA score were found to be normally distributed and are therefore presented as mean ± standard deviation. Categorical variables were expressed as frequencies and percentages. Statistical analyses were performed using IBM SPSS Statistics for Windows, Version 26.0 (Released 2018; IBM Corp., Armonk, NY, USA).

Group comparisons were conducted between predefined groups (survivors vs. non-survivors and MDR vs. non-MDR). Continuous variables were compared using the Student's t-test or the Mann-Whitney U test, and categorical variables using the chi-square test or Fisher's exact test, as appropriate.

Analyses were conducted at two complementary levels, depending on the research question and outcome of interest. Patient-based analyses (n = 80) were used for clinical characteristics, infection site classification, group comparisons (survivors vs. non-survivors; MDR vs. non-MDR), and outcome assessment. Isolate-based analyses (n = 142) were used for pathogen distribution and antimicrobial resistance profiling.

Infectious episodes were defined at the patient level, and only the first clinically significant isolate per patient per infectious episode was considered to avoid duplication bias. For polymicrobial infections, all clinically significant pathogens isolated from the same episode were retained, which explains why the total number of isolates (n = 142) exceeds the number of patients.

All tests were two-sided, and a p-value < 0.05 was considered statistically significant. No multivariable analysis was performed due to the limited sample size and the associated risk of model overfitting. Observed associations should therefore be interpreted as exploratory and hypothesis-generating only.

Ethical considerations

The study protocol was reviewed by the Institutional Review Board of Ibn Rochd University Hospital, Casablanca, Morocco, which waived the requirement for formal ethical approval due to the observational and non-interventional nature of the study using anonymized data collected during routine clinical care. The requirement for informed consent was waived. All procedures were conducted in accordance with institutional guidelines and the principles of the Declaration of Helsinki.

## Results

Results are presented using patient-based analyses for clinical characteristics and infection sites, and isolate-based analyses for microbiological distribution and antimicrobial resistance patterns. Because some patients had multiple infection sites or polymicrobial infections, percentages may exceed 100%.

Clinical profile of the study population

A total of 80 adult patients with sepsis or septic shock were included. The study population was predominantly male (50/80, 62%), with a high proportion of medical admissions (44/80, 55%) and hospital- or ICU-acquired infections (56/80, 70%).

At ICU admission, patients presented with marked severity, reflected by a mean SOFA score of 8 ± 4, and 33/80 (41%) met the criteria for septic shock. Exposure to major ICU-related risk factors was frequent, including mechanical ventilation in 54/80 (68%) of patients, central venous catheterization in 60/80 (75%), and prior antibiotic exposure in 48/80 (60%).

Baseline characteristics of the study population are summarized in Table 1.

**Table 1:** Baseline characteristics of the study population (n = 80) Values are presented as mean ± SD or n (%). ICU: intensive care unit, LOS: length of stay.

Variable	Value
Age (years), mean ± SD	50 ± 16.5
Male sex, n (%)	50 (62%)
Comorbidities, n (%)	
Hypertension	30 (38%)
Diabetes mellitus	26 (32%)
Chronic kidney disease	12 (15%)
Malignancy	8 (10%)
Type of admission, n (%)	
Medical	44 (55%)
Surgical	36 (45%)
Origin of infection, n (%)	
Community-acquired	24 (30%)
Hospital- or ICU-acquired	56 (70%)
Severity at ICU admission	
SOFA score, mean ± SD	8 ± 4
Septic shock, n (%)	33 (41%)
Healthcare-related risk factors, n (%)	
Mechanical ventilation	54 (68%)
Central venous catheter	60 (75%)
Prior antibiotic exposure (≤90 days)	48 (60%)
Clinical outcomes	
ICU LOS before infection (days), median	5
ICU mortality, n (%)	36 (45%)

Peritonitis was the most frequent infection site, identified in 49 patients (61%), of whom 42 (85.7%) were surgical admissions and 7 (14.3%) were medical admissions. The main etiologies included digestive perforation (17/49, 34.7%), anastomotic leak (12/49, 24.5%), postoperative intra-abdominal abscess (6/49, 12.2%), complicated appendicitis (5/49, 10.2%), infected pancreatitis (3/49, 6.1%), mesenteric ischemia (3/49, 6.1%), and other causes (3/49, 6.1%).

This distribution likely reflects the case-mix of our tertiary care center, which serves as a referral hospital for complex surgical emergencies. The remaining infection sites included bloodstream infection in 15 patients (19%) and pulmonary infection in 9 patients (11%). Clinical infection sites and associated microbiological distribution are summarized in Table 2.

**Table 2:** Clinical infection sites and associated microbiological distribution Values are expressed as number (percentage). Only pathogens accounting for ≥9% of isolates per site are listed.

Infection site	Patients, n (%)	Isolates (n)	Main pathogens (n, %)
Peritonitis	49 (61%)	32	*E. coli* (12, 37.5%), *P. aeruginosa* (6, 18.7%), *Enterococcus* spp. (5, 15.6%), *Enterobacter* spp. (4, 12.5%), *Bacteroides* spp. (3, 9.4%), *Acinetobacter* spp. (2, 6.3%)
Bloodstream infection	15 (19%)	52	*Acinetobacter* spp. (18, 34.6%), *Staphylococcus* spp. (13, 25%), *E. coli* (9, 17.3%), *P. aeruginosa* (7, 13.5%)
Pulmonary infection	9 (11%)	42	*Acinetobacter* spp. (12, 28.6%), *P. aeruginosa* (9, 21.4%), *Enterobacter* spp. (5, 11.9%), *Staphylococcus* spp. (4, 9.5%)
Urinary infection	9 (11%)	10	*Enterobacter *spp. (4, 40%), *E. coli* (4, 40%)
Catheter-related infection	6 (7%)	6	*Staphylococcus* spp. (5, 83.3%)

Sample distribution included 96 blood cultures, 48 respiratory samples, 24 peritoneal samples, 14 urinary samples, and 10 catheter-related samples, with positivity rates of 54.2%, 75%, 91.7%, 71.4%, and 60%, respectively.

Microbiological profile 

Among the 142 isolates, *Acinetobacter* spp. accounted for 28/142 (19.7%), *Escherichia coli* for 26/142 (18.3%), and *Pseudomonas aeruginosa* for 20/142 (14.1%). Gram-positive cocci included *Staphylococcus aureus* (7/142, 4.9%) and coagulase-negative staphylococci (11/142, 7.7%). Other organisms included *Enterobacter* spp. (12/142, 8.5%), *Enterococcus* spp. (8/142, 5.6%), and anaerobes such as *Bacteroides* spp. (6/142, 4.2%). Other organisms accounted for 24/142 isolates (16.9%). Among blood culture isolates (n = 52), *Acinetobacter* spp. accounted for 18/52 (34.6%), *Staphylococcus* spp. for 13/52 (25%), *E. coli* for 9/52 (17.3%), and *P. aeruginosa* for 7/52 (13.5%).

Among respiratory isolates (n = 42), *Acinetobacter* spp. accounted for 12/42 (28.6%), *P. aeruginosa* for 9/42 (21.4%), *Enterobacter* spp. for 5/42 (11.9%), and *Staphylococcus* spp. for 4/42 (9.5%).

Among peritoneal isolates (n = 32), *E. coli* accounted for 12/32 (37.5%), *P. aeruginosa* for 6/32 (18.7%), *Enterococcus* spp. for 5/32 (15.6%), *Enterobacter* spp. for 4/32 (12.5%), anaerobes including *Bacteroides* spp. for 3/32 (9.4%), and *Acinetobacter* spp. for 2/32 (6.3%).

**Figure 1:**
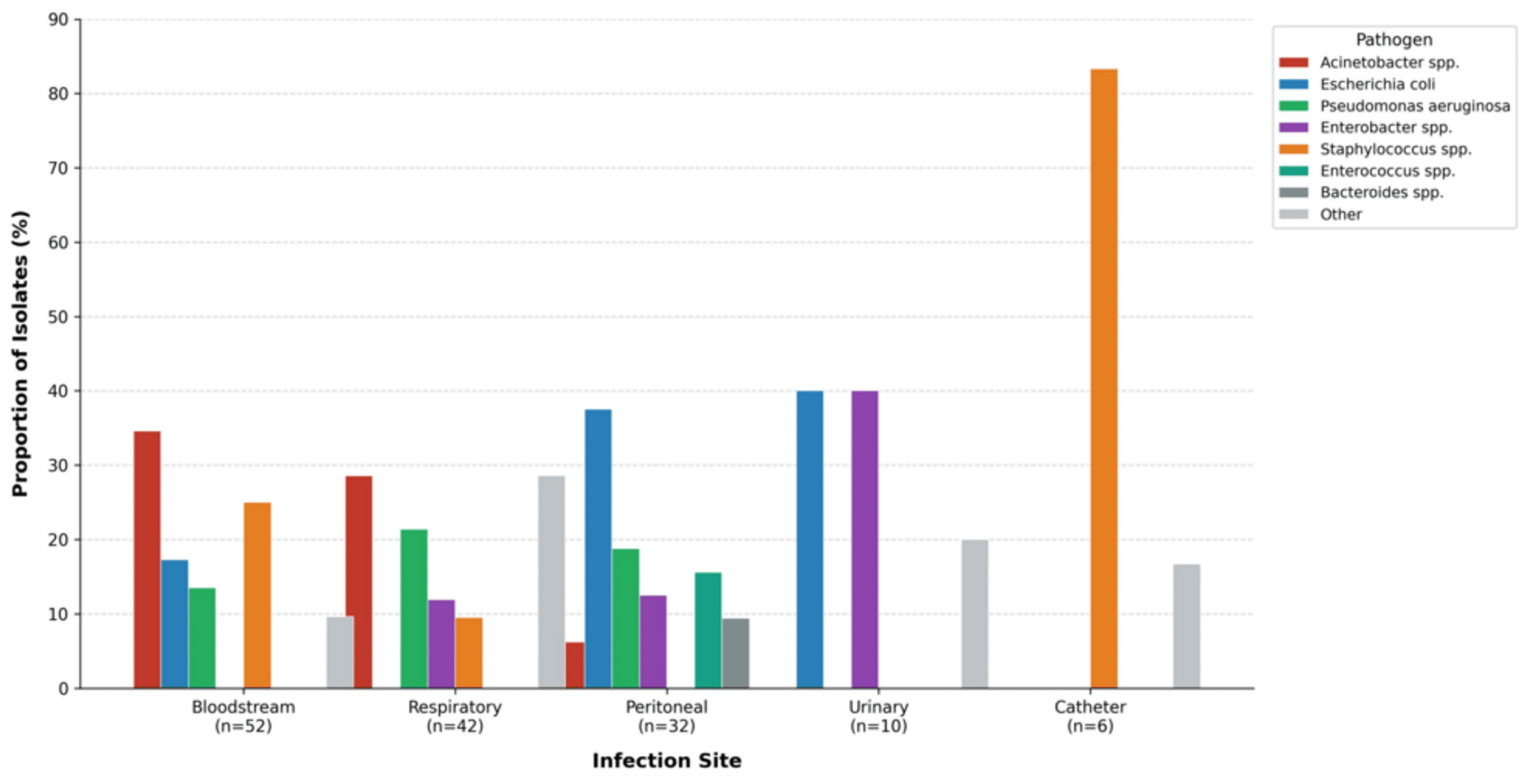
Distribution of major pathogens by infection site Values represent the proportion (%) of each pathogen among total isolates per infection site.

Urinary isolates (n = 10) included *Enterobacter* spp. (4/10, 40%) and *E. coli* (4/10, 40%), whereas catheter-related isolates (n = 6) included *Staphylococcus* spp. (5/6, 83.3%). The distribution of major pathogens by infection site is illustrated in Figure 1.

Microbiological sampling 

A total of 192 microbiological samples were collected from 80 patients, of which 126 (65.6%) were positive, yielding 142 clinically significant isolates. The higher number of isolates relative to the number of patients and positive samples reflects the occurrence of polymicrobial infections, which were identified in 22/80 patients (27.5%).

Sample distribution included 96 blood cultures, 48 respiratory samples, 24 peritoneal samples, 14 urinary samples, and 10 catheter-related samples, with positivity rates of 52/96 (54.2%), 36/48 (75%), 22/24 (91.7%), 10/14 (71.4%), and 6/10 (60%), respectively.

Of the 80 included patients, 64 (80.0%) contributed two or more microbiological samples, reflecting routine ICU diagnostic practice. This high sampling rate strengthens the reliability of microbiological documentation in this cohort.

In Table 2, the number of isolates per sample type reflects all positive cultures obtained from each source across the cohort, regardless of the primary infection site classification. Patients with a primary infection site other than the bloodstream may also have yielded positive blood cultures, accounting for secondary bacteremias.

Multidrug resistance

MDR was identified in 58/142 isolates (40.8%) and in 36/80 patients (45%). *Acinetobacter* spp. showed the highest MDR rate at 22/28 cases (78.6%), followed by *Enterobacter* spp. at 6/12 (50%), *P. aeruginosa* at 9/20 (45%), and *E. coli* at 10/26 (38.5%).

Antimicrobial resistance patterns

In *Acinetobacter* spp., resistance rates were 20/26 (76.9%) for ciprofloxacin, 20/23 (87.0%) for gentamicin, and 21/27 (77.8%) for imipenem. Colistin resistance remained low at 3/28 (10.7%). Antimicrobial resistance patterns for the main bacterial species are detailed in Table 3. Resistance rates are presented at the isolate level, with the number of resistant isolates reported as n/N (%), where N corresponds to the number of isolates tested for each antibiotic.

**Table 3:** Antimicrobial resistance patterns of major bacterial species Values are expressed as n/N (%). Dashes (-) indicate the antibiotics are not routinely tested for this species. R: resistance.

Species	n isolates	Ciprofloxacin R, n/N (%)	Gentamicin R, n/N (%)	Imipenem R, n/N (%)	Colistin R, n/N (%)
*Acinetobacter* spp.	28	20/26 (76.9%)	20/23 (87.0%)	21/27 (77.8%)	3/28 (10.7%)
*Escherichia* coli	26	10/26 (38.5%)	7/26 (26.9%)	1/26 (3.8%)	0/26 (0%)
Pseudomonas aeruginosa	20	9/20 (45.0%)	8/20 (40.0%)	7/20 (35.0%)	0/20 (0%)
*Enterobacter* spp.	12	5/12 (41.7%)	4/12 (33.3%)	2/12 (16.7%)	0/12 (0%)
Staphylococcus aureus	7	-	2/7 (28.6%)	-	-
Coagulase-negative staphylococci	11	-	5/11 (45.5%)	-	-
*Enterococcus* spp.	8	-	-	-	2/8 (25.0%)

Clinical outcomes and factors associated with mortality

ICU mortality was 36/80 (45%). ICU mortality was higher in patients with MDR infection than in those without MDR pathogens (67% vs. 27%, p < 0.001). Acinetobacter spp. was more frequently isolated in non-survivors than in survivors (18/36 (50%) vs. 13/44 (30%), p = 0.040). Non-survivors had higher SOFA scores at ICU admission and a higher proportion of septic shock (p < 0.001 for both). No significant differences were observed for age, sex, or infection site. Clinical characteristics of survivors and non-survivors are compared in Table 4.

**Table 4:** Comparison of clinical characteristics between survivors and non-survivors Statistical analyses were performed using Student's t-test for continuous variables and chi-square test (or Fisher's exact test when expected cell count < 5) for categorical variables. Bold p-values indicate statistical significance (p < 0.05)

Variable	Survivors (n = 44)	Non-survivors (n = 36)	Test statistic	p-value
Age (years), mean ± SD	47 ± 15	54 ± 17	t = −1.931	0.080
Male sex, n (%)	26 (59%)	24 (67%)	χ² = 0.485	0.45
SOFA score at ICU admission, mean ± SD	6 ± 3	10 ± 4	t = −4.965	<0.001
Septic shock, n (%)	10 (23%)	23 (64%)	χ² = 13.843	<0.001
Peritonitis, n (%)	28 (64%)	21 (58%)	χ² = 0.235	0.56
Pulmonary infection, n (%)	4 (9%)	5 (14%)	χ² = 0.457	0.52
Urinary infection, n (%)	5 (11%)	4 (11%)	χ² = 0.001	0.95
Primary bloodstream infection, n (%)	7 (16%)	8 (22%)	χ² = 0.518	0.48
MDR infection, n (%)	12 (27%)	24 (67%)	χ² = 12.415	<0.001
Acinetobacter spp., n (%)	13 (30%)	18 (50%)	Fisher's exact	0.040

Clinical outcomes according to MDR status

Patients with MDR infections had a significantly higher SOFA score at ICU admission compared to those without MDR pathogens (9 ± 4 vs. 7 ± 3, p = 0.010). Septic shock was significantly more frequent in the MDR group (20/36 (56%) vs. 13/44 (30%), p = 0.020). ICU mortality was higher in patients with MDR infection than in those without MDR pathogens (24/36 (67%) vs. 12/44 (27%), p < 0.001). No significant differences were observed between the two groups regarding age, sex, or infection site. A detailed comparison is presented in Table 5.

**Table 5:** Comparison between patients with MDR and non-MDR infections Statistical analyses were performed using Student's t-test for continuous variables and chi-square test (or Fisher's exact test when expected cell count < 5) for categorical variables. Bold p-values indicate statistical significance (p < 0.05). ICU: intensive care unit, MDR: multidrug resistance.

Variable	MDR (n = 36)	Non-MDR (n = 44)	Test statistic	p-value
Age (years), mean ± SD	52 ± 17	48 ± 15	t = 1.103	0.22
Male sex, n (%)	22 (61%)	28 (64%)	χ² = 0.054	0.78
SOFA score at ICU admission, mean ± SD	9 ± 4	7 ± 3	t = 2.483	0.010
Septic shock, n (%)	20 (56%)	13 (30%)	χ² = 5.527	0.020
Peritonitis, n (%)	24 (67%)	25 (57%)	χ² = 0.809	0.34
Pulmonary infection, n (%)	5 (14%)	4 (9%)	χ² = 0.457	0.52
Urinary infection, n (%)	3 (8%)	6 (14%)	χ² = 0.558	0.39
Primary bloodstream infection, n (%)	8 (22%)	7 (16%)	χ² = 0.518	0.48
ICU mortality, n (%)	24 (67%)	12 (27%)	χ² = 12.415	<0.001

## Discussion

In this prospective single-center ICU study, our findings provide a nuanced characterization of ICU-specific microbial ecology by demonstrating three key elements: a predominance of Gram-negative bacteria, a prominent and clinically significant role of *Acinetobacter* spp., and a marked heterogeneity in pathogen distribution across infection sites. This heterogeneity suggests that ICU sepsis represents a spectrum of infections with distinct microbiological profiles and therapeutic implications, rather than a single homogeneous entity.

A key finding of this study is the dual epidemiological pattern observed in our ICU. On one hand, intra-abdominal infections, predominantly driven by *E. coli* and polymicrobial flora, represent a major source of sepsis. On the other hand, bloodstream and respiratory infections are largely dominated by *Acinetobacter* spp., reflecting a strong influence of ICU-acquired infections and hospital microbial ecology. This distinction highlights the need for site-adapted empirical antibiotic strategies and supports a more individualized approach to infection management in the ICU.

The predominance of Gram-negative bacteria observed in our cohort is consistent with reports from ICUs in low- and middle-income countries, where non-fermenting Gram-negative bacilli, such as *Acinetobacter baumannii* and *P. aeruginosa,* are frequently identified as leading pathogens in severe infections [8-10]. In contrast, studies from high-income settings often report a relatively higher proportion of Gram-positive organisms, reflecting differences in ICU ecology, antimicrobial exposure, and infection control practices [11].

A major and clinically relevant finding of this study is the prominent role of *Acinetobacter* spp., particularly in bloodstream and respiratory infections, with very high rates of MDR. Carbapenem-resistant *A. baumannii* has been identified as a critical-priority pathogen by the WHO due to its global dissemination and limited therapeutic options [12]. Our findings are consistent with previous studies reporting high resistance rates and an association with poor clinical outcomes [8-10]. The predominance of *Acinetobacter* and the high burden of MDR organisms in our cohort likely reflect the interaction between ICU-related risk factors and microbial ecology, including severity of illness, invasive device exposure, and prior antibiotic use, well-established drivers of healthcare-associated infections [5]. Several factors may further explain this predominance: *Acinetobacter* is known for its ability to survive in the hospital environment and persist on surfaces and medical equipment, facilitating cross-transmission among critically ill patients [13]. In addition, ICU patients are frequently exposed to invasive procedures, prolonged hospitalization, mechanical ventilation, and repeated courses of broad-spectrum antibiotics, all of which contribute to the selection and persistence of MDR organisms.

Another notable finding is the high proportion of peritonitis, which was the leading source of infection in our cohort, identified in 61% of patients. Among these, 42/49 patients (85.7%) were surgical admissions, with digestive perforation (34.7%), anastomotic leak (24.5%), and postoperative intra-abdominal abscess (12.2%) as the most frequent etiologies. Intra-abdominal infections remain a major cause of severe sepsis, particularly in surgical ICUs, and are typically polymicrobial [13]. The predominance of peritonitis in our study likely reflects the predominantly surgical case-mix of our tertiary care center, which serves as a referral hospital for complex abdominal surgical emergencies in the Casablanca region. The coexistence of a high burden of intra-abdominal infections and a predominance of multidrug-resistant Gram-negative pathogens highlights the importance of integrating both infection source and healthcare-associated risk in empirical antibiotic selection.

Marked interspecies variability in antimicrobial resistance was observed. *Acinetobacter* spp. exhibited high resistance across multiple antibiotic classes, including ciprofloxacin (20/26, 76.9%), gentamicin (20/23, 87.0%), and imipenem (21/27, 77.8%), while colistin resistance remained low (3/28, 10.7%), suggesting that it may still represent a therapeutic option in selected cases. However, its use should be carefully balanced against its nephrotoxicity and guided by local antimicrobial stewardship policies.

When stratified by infection site, resistance rates of *Acinetobacter* spp. were broadly similar between bloodstream and respiratory isolates: ciprofloxacin (13/16, 81.3% vs. 7/10, 70.0%), gentamicin (12/14, 85.7% vs. 6/7, 85.7%), imipenem (13/16, 81.3% vs. 7/9, 77.8%), and colistin (2/16, 12.5% vs. 1/10, 10.0%). Although subgroup sizes preclude formal statistical comparison, these findings suggest consistently high resistance levels regardless of infection site.

In contrast, *E. coli* remained largely susceptible to carbapenems (1/26, 3.8% resistance), highlighting important interspecies differences in resistance profiles. These findings support a risk-adapted approach to empirical therapy based on local epidemiology, rather than uniform broad-spectrum strategies [11].

The association between MDR infections, *Acinetobacter* isolation, and increased mortality observed in our study highlights the clinical impact of antimicrobial resistance in critically ill patients. ICU mortality was significantly higher in patients with MDR infections compared to those without (67% vs. 27%, p < 0.001), and *Acinetobacter* spp. was more frequently isolated in non-survivors than in survivors (50% vs. 30%, p = 0.040).

Although causality cannot be definitively established due to the observational design, these findings underscore the potential clinical implications of antimicrobial resistance and support the rationale for antimicrobial strategies guided by local epidemiological data. However, the adequacy of empirical antibiotic therapy was not assessed in this study.

This study has several limitations. First, its single-center design and limited sample size (n = 80) may affect generalizability and precluded multivariable analysis; observed associations should therefore be interpreted as exploratory and hypothesis-generating only. Second, microbiological analysis was restricted to patients with documented isolates, which may introduce selection bias toward more severe or microbiologically documented infections. Third, extensively drug-resistant (XDR) organisms were not assessed, which may underestimate the full burden of antimicrobial resistance in our cohort.

Despite these limitations, this study provides valuable data from a North African ICU, a setting that remains underrepresented in the literature. Our findings highlight a distinct ICU ecological profile characterized by a high prevalence of multidrug-resistant Gram-negative bacteria, a dominant role of *Acinetobacter* spp., and significant site-related heterogeneity in pathogen distribution. These results emphasize the importance of continuous local microbiological surveillance and support the implementation of ICU-specific and infection site-adapted empirical antibiotic strategies.

## Conclusions

In this prospective North African ICU study, sepsis was characterized by a high burden of multidrug-resistant Gram-negative bacteria, with *Acinetobacter* spp. emerging as the dominant pathogen in bloodstream and respiratory infections, reaching MDR rates of 78.6%. A key finding of this study is the marked heterogeneity in pathogen distribution across infection sites, suggesting that ICU sepsis may be better understood as a heterogeneous spectrum of infections rather than a single homogeneous entity, thereby reinforcing the need for site-adapted and ecology-driven empirical antibiotic strategies. The strong association between MDR infections and ICU mortality (67% vs. 27%, p < 0.001) highlights the potential clinical impact of antimicrobial resistance in critically ill patients and supports the importance of antimicrobial strategies guided by local ecological data. These findings support the importance of continuous local microbiological surveillance and the implementation of ICU-specific empirical antibiotic strategies. Further prospective multicenter studies are needed to validate these findings and assess the impact of ecology-driven antibiotic strategies on treatment adequacy and clinical outcomes in North African ICUs.

## References

[1] Rudd KE, Johnson SC, Agesa KM (2020). Global, regional, and national sepsis incidence and mortality, 1990-2017: analysis for the Global Burden of Disease Study. Lancet.

[2] Seymour CW, Liu VX, Iwashyna TJ (2016). Assessment of clinical criteria for sepsis: for the third international consensus definitions for sepsis and septic shock (Sepsis-3). JAMA.

[3] Vincent JL, Sakr Y, Sprung CL (2006). Sepsis in European intensive care units: results of the SOAP study. Crit Care Med.

[4] Rhodes A, Evans LE, Alhazzani W (2017). Surviving sepsis campaign: international guidelines for management of sepsis and septic shock: 2016. Intensive Care Med.

[5] Kollef MH, Shorr AF, Bassetti M, Timsit JF, Micek ST, Michelson AP, Garnacho-Montero J (2021). Timing of antibiotic therapy in the ICU. Crit Care.

[6] Singer M, Deutschman CS, Seymour CW (2016). The third international consensus definitions for sepsis and septic shock (Sepsis-3). JAMA.

[7] Magiorakos AP, Srinivasan A, Carey RB (2012). Multidrug-resistant, extensively drug-resistant and pandrug-resistant bacteria: an international expert proposal for interim standard definitions for acquired resistance. Clin Microbiol Infect.

[8] Cavallo JD, Fabre R, Leblanc F (2002). Antibiotic susceptibility of Acinetobacter strains isolated from patients with nosocomial infections in ICU. J Antimicrob Chemother.

[9] Russo A, Bassetti M, Ceccarelli G (2019). Bloodstream infections caused by carbapenem-resistant Acinetobacter baumannii: Clinical features, therapy and outcome from a multicenter study. J Infect.

[10] Itani DM, Zahreddine N, Rajha E (2022). Risk factors for multidrug-resistant organism infections in critically ill patients. Antimicrob Resist Infect Control.

[11] Timsit JF, Bassetti M, Cremer O (2019). Rationalizing antimicrobial therapy in the ICU: a narrative review. Intensive Care Med.

[12] Tacconelli E, Carrara E, Savoldi A (2018). Discovery, research, and development of new antibiotics: the WHO priority list of antibiotic-resistant bacteria and tuberculosis. Lancet Infect Dis.

[13] Sartelli M, Weber DG, Ruppé E (2016). Antimicrobials: a global alliance for optimizing their rational use in intra-abdominal infections (AGORA). World J Emerg Surg.

